# Terahertz Vibrational Fingerprints Detection of Molecules with Particularly Designed Graphene Biosensors

**DOI:** 10.3390/nano12193422

**Published:** 2022-09-29

**Authors:** Xiaobing Han, Xueqin Shen, Yuanguo Zhou, Lin Wang, Qiang Ren, Yijun Cai, Reza Abdi-Ghaleh

**Affiliations:** 1 College of Communication and Information Engineering, Xi’an University of Science and Technology, Xi’an 710054, China; 2 School of Electronics and Information Engineering, Beihang University, Beijing 100191, China; 3 Fujian Provincial Key Laboratory of Optoelectronic Technology and Devices, Xiamen University of Technology, Xiamen 361024, China; 4 Department of Laser and Optical Engineering, University of Bonab, Bonab 5551761167, Iran

**Keywords:** terahertz biosensors, graphene, metasurface, surface plasmons, molecular vibrational fingerprints

## Abstract

In this research, an arc I-shaped graphene sensing structure with multi-resonance characteristics is proposed for the simultaneous detection of vibrational fingerprints with spectral separation in the terahertz range. The resonant frequencies of the sensor can be dynamically tuned by changing the gate voltage applied to the graphene arrays. The two vibrational fingerprints of lactose molecules (0.53 THz and 1.37 THz) in the transmission spectrum can be enhanced simultaneously by strictly optimizing the geometrical parameters of the sensor. More importantly, these two resonant frequencies can be tuned precisely to coincide with the two standard resonances of the lactose molecule. The physical mechanism of the sensor is revealed by inspection of the electric field intensity distribution, and the advantage of the sensor, which is its ability to operate at a wide range of incident angles, has been demonstrated. The sensing performance of the structure as a refractive index sensor has also been studied. Finally, a double arc I-shaped graphene sensor is further designed to overcome the polarization sensitivity, which demonstrates excellent molecular detection performance under different polarization conditions. This study may serve as a reference for designing graphene biosensors for molecular detection.

## 1. Introduction

In recent years, terahertz (THz) electromagnetic waves have attracted wide attention in molecular recognition due to their unique characteristics of low energy, non-ionization and fingerprint spectrum [1,2]. Since lactose molecules, nucleic acids and DNA have corresponding vibrational fingerprint spectra in the THz range [3], then the THz waves have natural advantages in the field of biomolecular detection [4]. By using THz spectroscopy, label-free and zero-contact detection of biomolecules can be achieved [5,6]. However, due to the weak interaction between THz waves and biomolecules, the sensitivity and accuracy of this technique for molecular detection are limited [7]. In addition, in the THz range, the sensing structure based on the planar metal is difficult to be used for THz sensing because the surface plasmon waves on the planar metal are not limited [8]. Moreover, the plasmon resonance wavelength of the metal surface is difficult to dynamically tune. To solve the above problems, a more efficient and accurate THz detection technology is urgently needed to be developed.

The resonant coupling between the plasma mode of the sensor and the characteristic mode of the molecule can be explained by the basic concept of hybridization-induced transparency (HIT). In HIT, the induced transparency can be achieved in a hybrid system consisting of a plasma structure and an atomic heterogeneity [9]. If the interaction between the plasma and the molecular system can be controlled so that the broadband oscillations in the plasma structure interact with the narrowband oscillations in the atomic system, resulting in destructive interference, then induced narrowband transparency within the broadband absorption lines can be observed [10]. Similarly, the sensor acts as a plasma structure with broadband absorption lines, while the molecule acts as an atomic system with very narrowband absorption lines. Resonant coupling occurs when the characteristic frequencies of the molecules overlap spectrally with the plasma resonance of this sensor. Thus, narrow transmittance peaks in a wider transmittance inclination can be considered as molecular signature fingerprints. This is the principle of sensing using this sensor.

Graphene is a two-dimensional monolayer of carbon atoms arranged in a honeycomb lattice [11]. It has a symmetric cone zero band gap structure and high carrier mobility, showing unique mechanical, optical, and electrical properties [12,13,14,15]. In the THz region, highly doped graphene has metal-like properties [16,17] which can generate high local surface plasmon polaritons (SPPs) [18,19], showing strong field confinement, dynamic tunability and high surface volume ratio [20,21,22]. Therefore, graphene-based optical biosensors usually have the advantages of high detection sensitivity and adjustable spectral selectivity. Graphene surface plasmon has extremely strong field constraints and low loss optical characteristics, while traditional precious metals do not. In recent years, researchers have tried to use graphene to improve the detection performance of sensing structures. In 2004, researchers from the IBM Corporation utilized graphene nanoribbons to detect surface-adsorbed thin films of polymer for the first time [23]. They further investigated the coupling between graphene plasmons and the vibrations of solid- and gas-phase molecules [24]. Rodrigo designed a biosensor based on graphene plasma resonance [25], which significantly enhanced the vibrational fingerprint signal of proteins. The plasma resonance frequency of the sensing structure was dynamically tuned by changing the gate voltage applied to the graphene nanoribbon to accurately detect the different vibration fingerprints of the protein. Hu reported a graphene plasma sensing structure designed on CaF${}_{2}$ nanofilm [26], which avoids the strong phonons produced by SiO${}_{2}$ and strong hybridization of graphene plasma, realizing the sensing of polymers and fingerprints of gases. Wu proposed a graphene-based biosensor that improved the performance of a conventional surface plasmon resonance (SPR) sensing structure by covering a single layer of graphene on a gold film [27]. Zhu et al. designed a hybrid metasurface with suspended graphene and gold nanoantennas; when the nanoantennas were deposited close enough (about 10 nm), ultrasensitive biosensing ability was achieved to probe low-molecular-weight analytes [28]. The strong adsorption capacity of graphene on biomolecules was also utilized to enhance the sensing performance of the sensing structure on biomolecules and accurately identified the vibration fingerprint of the detected molecules. However, all of these graphene-based single-resonance biosensors are not able to simultaneously recognize the multiple vibrational fingerprints in the same detection object. Therefore, the single resonance biosensor often produces incorrect positives when detecting objects with multiple vibration fingerprints [29]. To overcome this phenomenon, Cai and Mao reported the use of graphene plasmons for realizing multi-resonance sensing [30,31]. However, there are still shortcomings in polarization dependence, and the resonance peaks cannot precisely overlap with the molecular fingerprints.

In this paper, a multi-resonance tunable sensor based on an arc I-shaped graphene structure (AIGS) is proposed, which can be used to simultaneously detect multiple vibration fingerprint spectra in the THz range. By altering the gate voltage applied to the graphene surface, the resonant frequency of the structure can be dynamically tuned to the vibrational fingerprints of the lactose molecule. Through optimizing the geometric parameters, the two vibration fingerprints (0.53 THz and 1.37 THz) of lactose in the transmission spectrum can be simultaneously enhanced, and the corresponding resonant frequencies can be independently adjusted by varying the geometrical parameters. We also study the effect of incidence angle on the detection effect and the refractive index sensing performance of the sensor. In addition, to ensure its excellent molecular detection performance under different polarized incident lights, we further designed a quadruple rotationally symmetric double arc I-graphene structure (DAIGS) biosensor, analyzed the effect of the different incident angles and polarizations on the structure’s performance, and revealed its physical mechanism by investigating the electric field intensity distribution. Numerical results verify the efficiency and accuracy of the proposed two structures and their feasibility in biosensing applications.

## 2. Modeling and Methods

**Figure 1:**
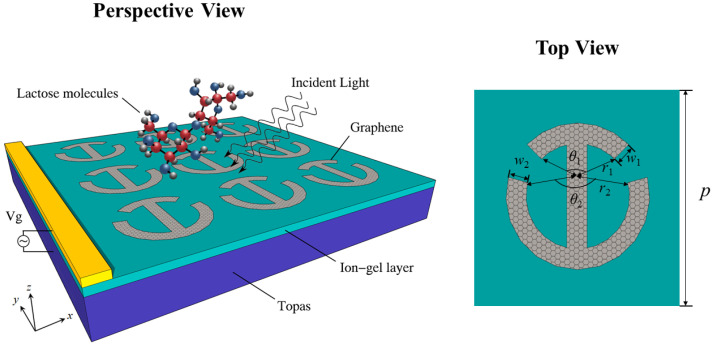
Schematic diagram of sensor based on arc I-graphene structure (AIGS).

Figure 1 describes the schematic diagram of the proposed arc I-shaped graphene structure (AIGS) for THz biomolecular detection. The geometrical parameters of the structure are *p* = 50 μm, $r_{1}$ = 14.6 μm, $w_{1}$ = 1.5 μm, $r_{2}$ = 20 μm, $w_{2}$ = 1.5 μm, $w_{3}$ = 1.5 μm, $\theta_{1}$ = 60${}^{\circ }$, and $\theta_{2}$ = 130${}^{\circ }$. The sensor consists of a cyclic olefin copolymer (Topas) substrate, an ion-gel layer, and a graphene-patterned layer. The relative permittivity of Topas is chosen as 2.35. To conveniently realize dynamic tuning of graphene Fermi energy, a high capacitance ion-gel film layer was added below the graphene pattern layer. The thickness of the ion-gel layer was set at 100 nm, and its relative permittivity was 1.82. We can make a gold grating contact on the ion-gel layer to act as an electrode for electrostatic doping. Monolayers of graphene could be grown by the Chemical Vapor Deposition (CVD) process and then transferred to an ion-gel [32]. Graphene sheets are patterned using electron beam lithography [33]. The interaction is investigated using COMSOL Multiphysics, which solves Maxwell equations with the finite element method (FEM) in the frequency domain. The THz waves are irradiated downward from the upper surface of the structure, and periodic boundary conditions are applied in the x- and y-axis directions. The graphene layer has the characteristics of a locally enhanced electromagnetic field, which requires a dense mesh size for simulation. Because graphene is very thin compared to the incident wavelength, it can be assumed that it is a two-dimensional surface [34]. According to the Kubo formula [35], the graphene conductivity can be calculated by the following expressions:

(1) $$\sigma \left(\omega ,E_{F},\Gamma ,T\right)=\sigma_{\mathrm{intra}}+\sigma_{\mathrm{inter}}$$

(2) $$\sigma_{\mathrm{intra}}=\frac{je^{2}}{\pi \hbar \left(\omega -j2\Gamma \right)}\int_{0}^{\infty }\xi \left(\frac{\partial f_{d}\left(\xi ,E_{F},T\right)}{\partial \xi }-\frac{\partial f_{d}\left(-\xi ,E_{F},T\right)}{\partial \xi }\right)d\xi$$

(3) σinter=−je2ω−j2Γπℏ2∫0∞fd−ξ,EF,T−fdξ,EF,Tω−j2Γ2−4ξξℏℏ2dξ

(4) fdξ,EF,T=eξ−EFξ−EFkBTkBT+1−1

where $\sigma_{\mathrm{intra}}$ and $\sigma_{\mathrm{inter}}$ are the intra-band and inter-band conductivity of graphene, respectively. ω is the angular frequency of the incident terahertz wave, $E_{F}$ is the Fermi energy of graphene, ξ is the electron energy, *e* is the electronic charge, *ℏ* is the reduced Planck constant, $k_{B}$ is the Boltzmann constant, T is the absolute temperature, Γ is the scattering rate, $2\Gamma =\tau^{-1}$ (τ is the relaxation time of the electron-phonon), and $f_{d}\left(\xi ,E_{F},T\right)$ is the Fermi–Dirac distribution.

In the THz region, since the incident photon energy is much smaller than the graphene Fermi energy, the effect caused by the inter-band transition is negligible, so the intra-band transition dominates the graphene conductivity. Assuming T is 300 K, the Kubo formula can be simplified as [36]:

(5) $$\sigma_{g}=\frac{ie^{2}E_{F}}{\pi \hbar^{2}\left(\omega +i\tau^{-1}\right)}$$

Therefore, the surface conductivity of graphene depends only on ω, $E_{F}$, and τ. In the processes of simulation, τ=1 ps [37], and the lactose molecular thickness was set to 0.1 μm. The relative permittivity $\varepsilon_{r}$ can be accurately described by the Lorentz–Drude model [38]:

(6) $$\varepsilon_{r}=\varepsilon_{0}\left[\varepsilon_{\infty }+\sum_{i=1}^{3}\frac{v_{pi}^{2}}{v_{i}^{2}-v^{2}-i\gamma_{i}v}\right]$$

where $\varepsilon_{0}$ is the vacuum permittivity, and $\varepsilon_{\infty }=3.2$ is the relative permittivity. $v_{1}=0.53$ THz, $v_{2}=1.195$ THz, and $v_{3}=1.37$ THz are the vibrational frequencies. $\gamma_{1}=21$ GHz, $\gamma_{2}=44$ GHz, and $\gamma_{3}=58$ GHz are the linewidths. $v_{p1}=0.123$ THz, $v_{p2}=0.072$ THz, and $v_{p3}=0.253$ THz are the corresponding plasma frequencies.

The lactose molecule has three vibrational fingerprints [39], namely $v_{1}$, $v_{2}$ and $v_{3}$. Because it shows strong resonance at 0.53 THz and 1.37 THz, these two characteristic frequencies are selected in this paper to verify the detection performance of the proposed sensing structure.

## 3. Results and Discussion

**Figure 2:**
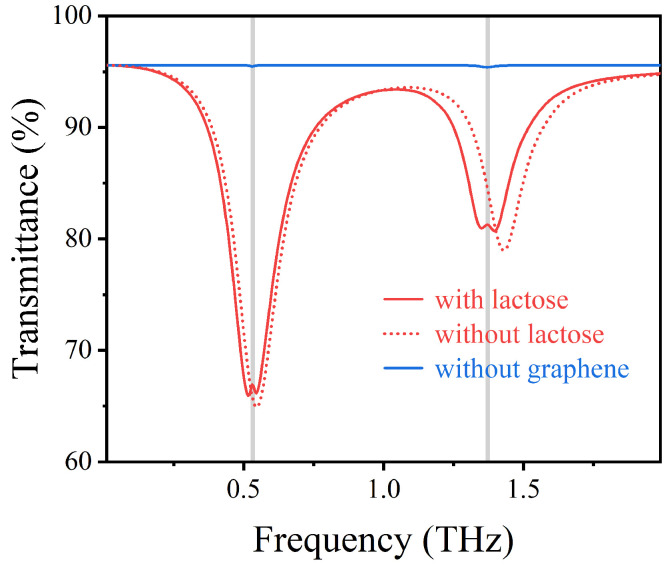
Transmission spectra of vertically incident TM waves with or without lactose AIGS.

To clarify the effect of AIGS on lactose fingerprints in the THz range, we calculated the transmission spectra of lactose molecules placed on exposed Topas substrates and AIGS with/without lactose coating. As shown in Figure 2, when lactose was placed on an exposed Topas substrate, its fingerprint feature showed a minimal valley in the transmission spectrum. Due to the weak interaction between lactose molecule and the THz waves, it is difficult to observe the fingerprint spectrum of lactose molecule directly. However, in the AIGS with lactose coverage (solid red curve) compared to non-lactose coverage (imaginary red curve), the electromagnetic environment on the structure surface changes, increasing the intensity of the electric field, which ultimately leads to a slight red shift in the resonant frequency of the sensing structure. In the sensing structure with lactose coverage ($E_{F}=0.66$ eV), the left resonance and right resonance of the structure coincide with the two vibration fingerprints (0.53 THz and 1.37 THz) of lactose molecules, respectively. The narrow transmission peak at lactose resonance is observed in the wide transmission resonance valley, which corresponds to the characteristic absorption of lactose. The occurrence of the narrow transmission peak indicates that the plasmon resonance produced by the structure is coupled with the vibration of the molecular bond in lactose.

**Figure 3:**
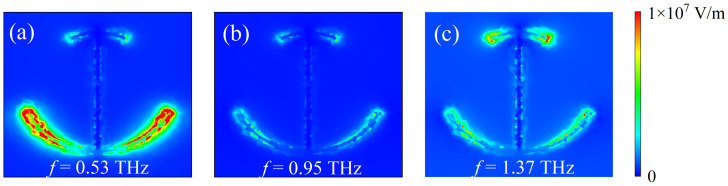
(**a**–**c**) Electric field distribution of AIGS covered with lactose at different frequencies under vertically incident TM waves.

To illustrate the resonant coupling characteristics of the AIGS sensor with the lactose molecule, Figure 3 depicts the electric field distribution of the sensing structure. When $E_{F}=0.66$ eV, there are two significant transmission valleys in the transmission spectrum near 0.53 THz and 1.37 THz. It can be seen from Figure 3a that at the resonant frequency f=0.53 THz, and the enhanced electric field is concentrated at the edge of the lower arc. This is because the incidence THz waves can excite graphene electrons to oscillate within a finite radian and thus excite local surface plasmon resonance (LSPR) in a larger graphene arc. As shown in Figure 3c, at resonant frequency f=1.37 THz, the locally enhanced electric field is mainly restrained at the edge of the upper arc. This is due to the strong LC resonance caused by the coupling of the incidence THz waves with the upper and lower arcs of the graphene, thus enhancing the local electromagnetic field. This resonance is efficient at capturing the energy of the THz waves and has enough time to eliminate the effects of ohmic losses within graphene [40]. Therefore, the electric field enhancement of the upper and lower arcs is helpful to increase the absorption cross-section of the lactose molecule, thus enhancing the vibration signal of the lactose molecule, which is conducive to the detection of a low concentration of lactose forming thinly onto the electrodes. As shown in Figure 3b, there is basically no electric field enhancement in the graphene arc I-shape at the non-resonant frequency (f=0.95 THz) because the non-resonant frequency is far away from the two plasmonic resonance frequencies of the structure.

**Figure 4:**
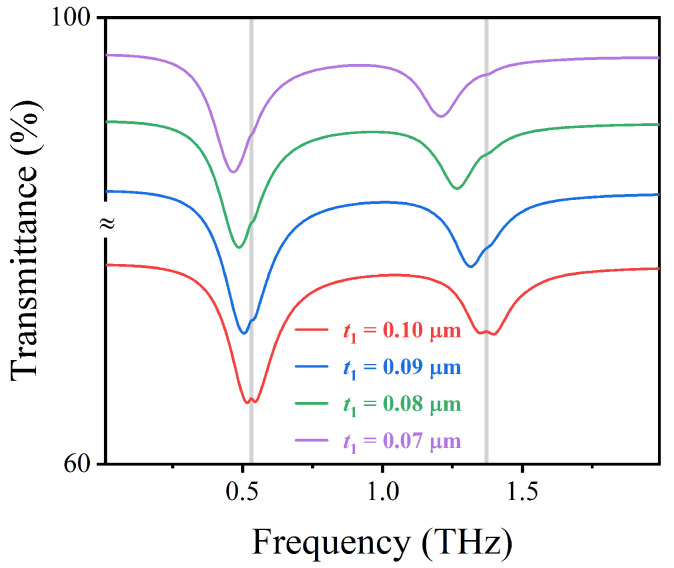
The transmission spectrum of AIGS at different lactose thicknesses $t_{1}$ under vertically incident TM waves.

Different thicknesses of lactose (0.10 μm, 0.09 μm, 0.08 μm, 0.07 μm) were covered on the designed sensor, and the fingerprint profile characteristics are shown in Figure 4. As the thickness of lactose decreases, the electromagnetic environment on the surface of the structure changes, resulting in a subtle red shift of the resonant frequency of the sensor. As shown in Figure 4, when the lactose thickness is close to 0.07 μm, an enhanced vibration signal can be faintly observed. Therefore, the detection limit of the sensor for the lowest concentration of lactose is 0.07 μm.

To further analyze the tunability of the proposed graphene sensor, the resonant frequency of the device is investigated by adjusting the Fermi energy of graphene by electric doping, which has the advantage of rapid tunability without changing the structure size. The relationship between the graphene Fermi energy $E_{F}$ and the gate voltage $V_{g}$ can be expressed as follows [41]:

(7) $$E_{F}=\hbar v_{f}\sqrt{\frac{\pi \varepsilon_{0}\varepsilon_{r}V_{g}}{et_{s}}}$$

where $v_{f}$ is Fermi velocity, $\varepsilon_{0}$ is the permittivity of free space, $\varepsilon_{r}$ is the relative permittivity of the spacer layer and $t_{s}$ is the thickness of the insulating layer.

**Figure 5:**
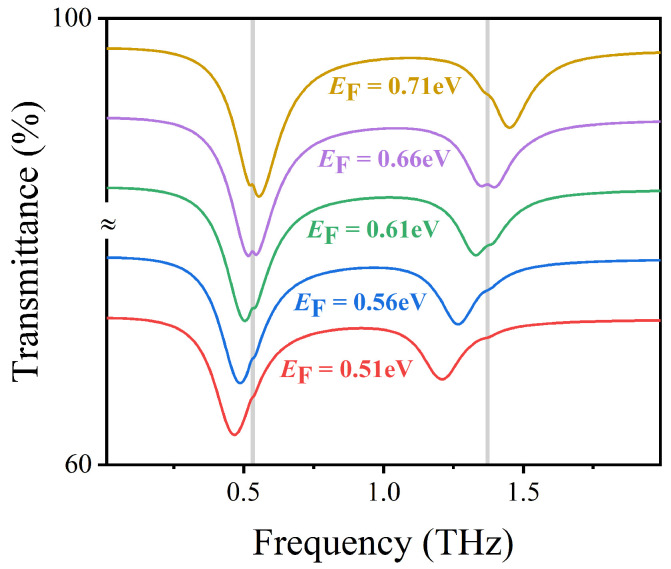
The transmission spectra of AIGS covered with lactose molecules at different Fermi energies under vertically incident TM waves.

Note that this characteristic makes up for the shortcoming that metal devices are not dynamically adjustable. Moreover, by controlling the gate voltage $V_{g}$, it is sensitive to detect the vibrational fingerprints of lactose molecules. As shown in Figure 5, we investigate the transmission spectrum variation of the sensor structure at different Fermi energy $E_{F}$. The grey vertical stripes at 0.53 THz and 1.37 THz represent two vibrational fingerprint spectra of lactose molecules. As $E_{F}$ decreases from 0.76 to 0.56 eV, the left resonant frequency red shifts from 0.56 to 0.46 THz, and the right resonant frequency redshifts from 1.46 to 1.21 THz. This is because the wave vector of the excited surface plasmon on graphene satisfies kspp∝ℏ2f2ℏ2f22e22e2EF, where *f* is the resonant frequency [42]. It follows that the resonant frequency is approximately proportional to $\sqrt{E_{F}}$, which explains that the reduction of Fermi energy $E_{F}$ can make the transmission spectrum red shift. The simulation results in Figure 5 also show that the position of the transmission spectrum can be dynamically tuned by changing the Fermi energy of graphene to conform to the vibrational fingerprints of lactose molecules without changing the structural parameters of the designed sensor. When $E_{F}=0.51$ eV, the two vibrational fingerprints of lactose are almost undetectable, because the formants are far from them. However, when $E_{F}=0.66$ eV, the left resonance and right resonance of the structure coincide with the vibration of lactose 0.53 THz and 1.37 THz, respectively, and the vibration signal of lactose is enhanced.

**Figure 6:**
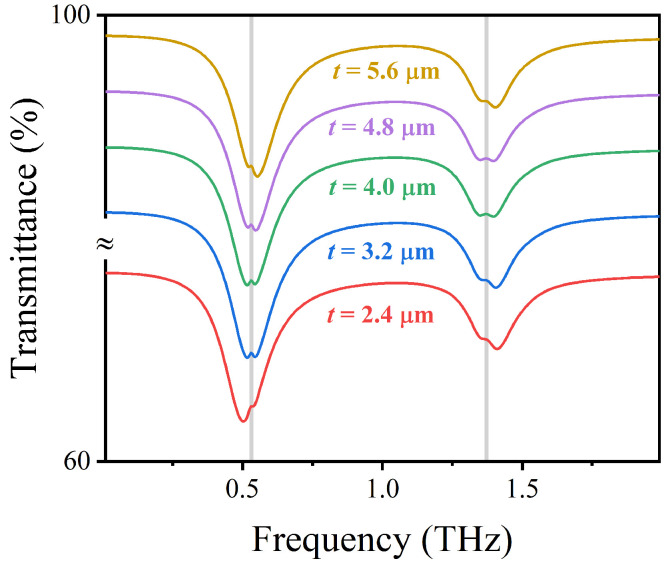
Transmission spectra of AIGS covered with lactose at different substrate thicknesses *t* under vertically incident TM waves.

Next, the influence of geometrical parameters of AIGS on the detection performance of sensing structures is studied. As the thickness *t* of the Topas substrate increases from 2.4 to 5.6 μm, the resonant frequency of the sensing structure has a slight blue shift and the transmission rate has a slight change. This is because the resonant frequency of the transmittance valley is mainly determined by the surface plasmon effect of graphene, but such subtle changes have a certain impact on the detection sensitivity of the sensing structure. As shown in Figure 6, the sensing structure can directly detect the two vibration fingerprints of lactose within the range of 2.4 to 5.6 μm of substrate Topas thickness, indicating that the sensing structure has good robustness to subtle changes in substrate Topas thickness. However, when the substrate Topas thickness is 4.0 μm, the plasma resonance of the sensing structure is strongly coupled with the vibration signal of lactose; that is, the detection performance of the sensing structure achieves the best effect, and the vibration signal of lactose is enhanced. Therefore, we selected *t* as 4.0 μm as the optimal thickness of substrate Topas.

**Figure 7:**
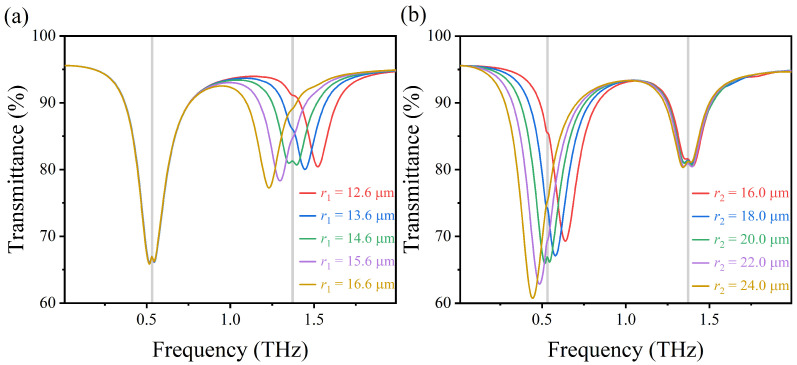
(**a**,**b**) Transmission spectra of AIGS covered with lactose at different arc radii $r_{1}$ and $r_{2}$ under vertically incident TM waves.

The plasma resonance of the sensing structure is highly dependent on the radius, widths, and radian of the upper and lower arcs in the structure. As shown in Figure 7a, by gradually adjusting the radius of the upper arc $r_{1}$ from 12.6 to 16.6 μm, the high-frequency transmission peak can be adjusted independently from 1.53 to 1.23 THz. As $r_{1}$ increases, the distance at the opening of the sensing structure gradually decreases, which leads to the enhancement of the induced LC resonance, and it further enhances the interaction between graphene and lactose molecule, so that the plasma frequency of the structure is strongly coupled with the characteristic absorption frequency of the lactose molecule. Similarly, as shown in Figure 7b, the low-frequency transmission peak can also be adjusted independently by changing the radius of the lower arc $r_{2}$. When radius $r_{2}$ increases, the resonant frequency shifts from 0.64 to 0.45 THz, which is mainly due to the increase of the effective resonant length at the edge of the lower arc. In summary, compared with $r_{2}$, a small change in $r_{1}$ will lead to a significant change in the effective resonant range of the high-frequency transmission peak and a corresponding enhancement of the vibration signal of lactose.

**Figure 8:**
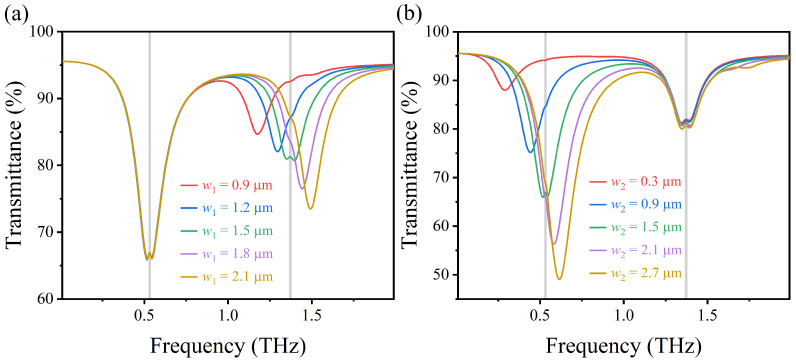
(**a**,**b**) Transmission spectra of AIGS covered with lactose at different arc widths $w_{1}$ and $w_{2}$ under vertically incident TM waves.

As shown in Figure 8a,b, as the width of upper and lower arcs $w_{1}$ and $w_{2}$ increase, not only the right resonant frequency and the left resonant frequency will be blue shifted correspondingly but also the filling ratio of graphene in the structure will be larger, which increases the energy dissipation in the graphene and leads to a decrease in the transmission rate and the amplitude of the transmission spectrum. That is because the effective refractive index of the excited LSPR on AIGS decreases with the increasing width of the upper and lower arc graphene, resulting in a decrease in the resonant wavelength and a significant blue shift in the corresponding resonant frequency.

**Figure 9:**
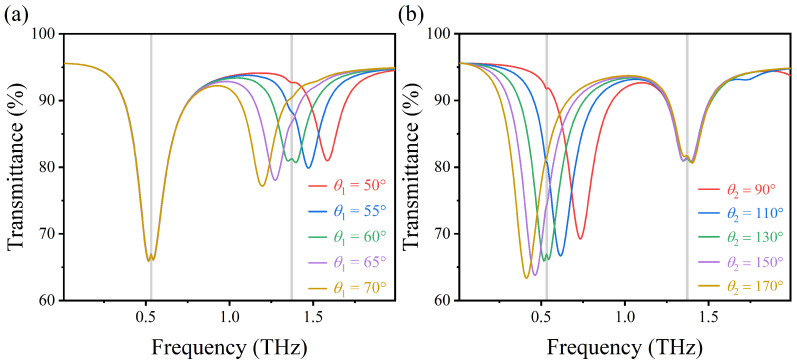
(**a**,**b**) Transmission spectra of AIGS covered with lactose at different radians $\theta_{1}$ and $\theta_{2}$ under vertically incident TM waves.

As shown in Figure 9a,b, by adjusting $\theta_{1}$ from 50° to 70° and $\theta_{2}$ from 90° to 170°, respectively, the right transmission peak shifted from 1.59 to 1.20 THz, and the left transmission peak shifted from 0.74 to 0.41 THz, both of which had red shifts. This is because with the increase of the effective radian of the sensing structure, the effective resonance length increases, and the binding performance of the LSPR electromagnetic field excited along the upper and lower graphene arcs increases, so the transmission frequency is red shifted. In addition, from the perspective of LC resonance, theoretically, the resonant frequency can be approximated as f=112πLCLC222πLCLC22∝11leffleff [43], where *L* and *C* represent the effective inductance and capacitance, respectively, and $l_{eff}$ represents the effective oscillation length of the resonator. The red shift of the left and right transmission peaks can be attributed to the increase of $l_{eff}$. Therefore, by changing parameters $\theta_{1}$ and $\theta_{2}$, the positions of the two transmission peaks of the structure can also be tuned to the vibration of the lactose.

The above simulation results show that the plasma resonance generated by the sensing structure can be strongly coupled with the vibration of the lactose molecules by independently adjusting the geometrical parameters of the sensor.

**Figure 10:**
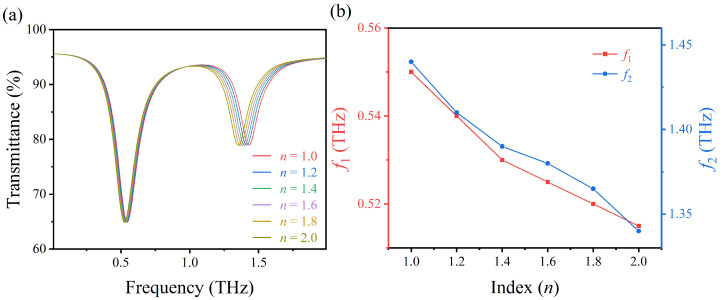
(**a**,**b**) Refractive index sensing characteristics of the AIGS.

To further study the refractive index sensing characteristics of the proposed structure, the transmission spectrum of AIGS in different refractive index layers are calculated. Since the refractive index of biomolecules is mostly in the range of 1.0–2.0, the refractive index of the material layer above AIGS is successively increased from 1.0 to 2.0 (step size is set as 0.2) in our calculation, and the thickness of the layer is fixed at 0.1 μm. The corresponding sensing structural parameters are set as *p* = 50 μm, $r_{1}$ = 14.6 μm, $w_{1}$ = 1.5 μm, $r_{2}$ = 20 μm, $w_{2}$ = 1.5 μm, $w_{3}$ = 1.5 μm, $\theta_{1}$ = 60${}^{\circ }$, $\theta_{2}$ = 130${}^{\circ }$, and $E_{F}=0.66$ eV. As shown in Figure 10a, with the increase of *n* from 1.0 to 2.0, both the left resonance frequency $f_{1}$ and the right resonance frequency $f_{2}$ have a red shift. The frequency shift of $f_{1}$ is 0.035 THz, and the frequency shift of $f_{2}$ is 0.1 THz. As shown in Figure 10b, with the change of refractive index *n* of the material layer, both left resonance frequency $f_{1}$ (red curve) and right resonance frequency $f_{2}$ (blue curve) show good linearity, which is beneficial to the practical application of refractive index sensing. To quantify the performance of the proposed sensor, we calculate the left and right resonance sensitivities S=ΔfΔfΔnΔn. The sensitivities of the left and the right transmission peaks are calculated to be 0.035 THz/RIU and 0.10 THz/RIU, respectively. The sensitivity of both the left and right resonance is lower than that of the sensor based on four metal crosses (0.45 THz/RIU) [44]. The sensitivity of the right resonance is higher than that of the sensor based on InSb-based metallic gratings for lactose molecular detection (0.06 THz/RIU) [45].

The performance of the proposed sensing structure under vertically incidence TM waves is investigated above. Then, we further study the impact of incidence angle θ on the AIGS performance. The other parameters are the same as those in Figure 10.

**Figure 11:**
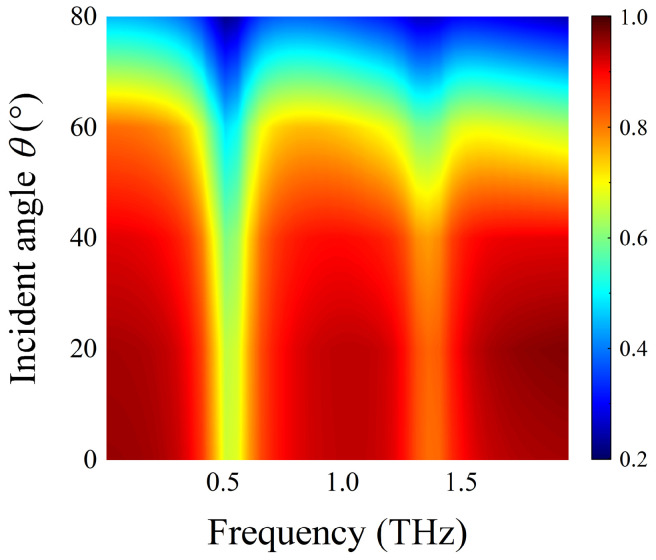
Transmission spectra of the AIGS covered with lactose at different incidence angles θ under TM polarization.

As shown in Figure 11, we exhibited the transmission spectra of the sensing structure covered by lactose molecules when the THz waves at different incidence angles θ irradiated to its surface. It can be observed that the resonance dip of AIGS is almost independent of incidence angle, and the transmission curve of the sensing structure maintains good stability. Especially, when the incidence angle θ ranges from 0° to 70°, there are two obvious resonance dips at 0.52 THz and 1.36 THz, which coincide with the two vibrational fingerprints of the lactose molecule in the spectrum. It demonstrates that the detection performance of AIGS at oblique incidence still has excellent robustness; thus, the proposed structure has a broad application prospect.

## 4. Double Arc I-Shape Graphene Sensing Structure

**Figure 12:**
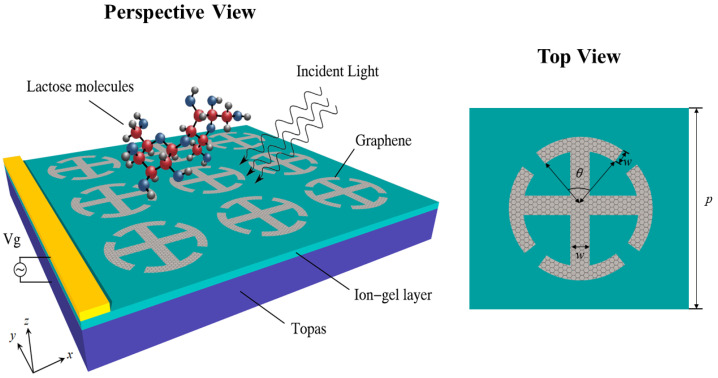
Schematic diagram of a double arc I-graphene structure (DAIGS).

**Figure 13:**
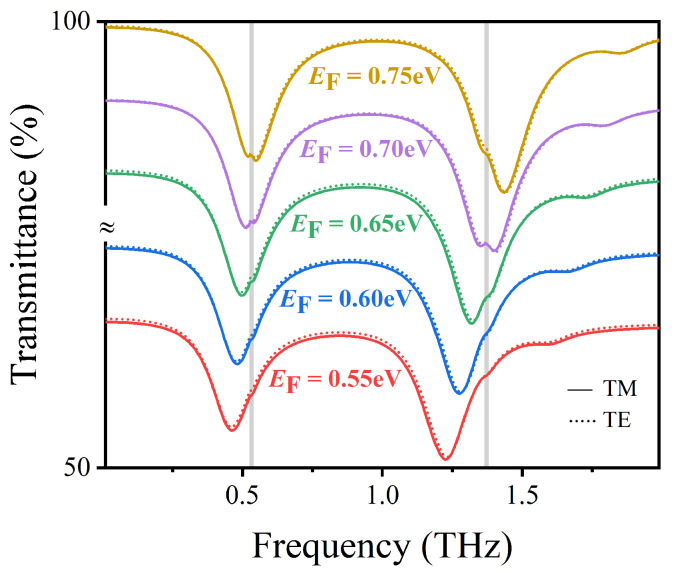
Transmission spectra of lactose covered DAIGS at different Fermi energies under TE or TM incidence waves.

In practical application, polarization insensitivity is helpful to improve detection efficiency and reduce the detection error caused by electromagnetic wave polarization. To address the problem of polarization sensitivity, we further propose a quadruple rotationally symmetric double arc I-graphene structure (DAIGS), as shown in Figure 12. The sensing structure also consists of a Topas substrate, an ion-gel layer, and a graphene pattern layer. By strictly optimizing the sensing structure, the corresponding geometric parameters were selected as p=38 μm, $r_{1}=12$ μm, w=1.5 μm, and $\theta =69^{\circ }$. The thickness of the Topas substrate and ion-gel layer is the same as in Figure 1. The influence of the THz waves in TM and TE polarization on DAIGS transmission spectra is depicted in Figure 13.

It can be seen that by adjusting $E_{F}$ from 0.55 to 0.75 eV, the left resonance frequency is blue-shifted from 0.46 to 0.55 THz, and the right resonance frequency is blue-shifted from 1.23 to 1.44 THz. We further scanned two vibrational fingerprint signals in lactose at 0.53 THz and 1.37 THz. When $E_{F}=0.70$ eV, the left and right resonances of the sensor structure coincide with the two vibration signals of lactose, which makes the vibration signals of lactose significantly enhanced. Therefore, this parameter is conducive to the detection of lactose molecules. Under TM and TE polarization, the soild and imaginary curves basically overlap with no significant difference, indicating that there is no difference in the sensing performance between TM and TE polarization, which is mainly because the 3D sensing unit structure is quadruple rotationally symmetric, as shown in Figure 13.

**Figure 14:**
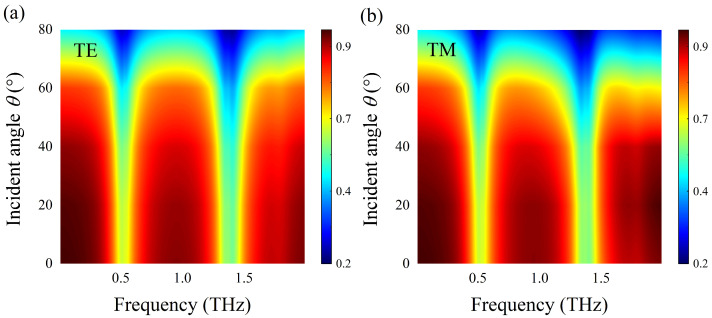
(**a**,**b**) Transmission spectra of lactose-covered DAIGS at different incidencce angle θ under TE or TM incidence waves.

Figure 14a,b, respectively, describe the transmission spectra of DAIGS covered with lactose at different incidence angles under TE and TM polarization, where the *x*-axis represents frequency and the *y*-axis represents incidence angle θ. The Fermi energy of graphene is fixed at $E_{F}=0.70$ eV. It is noteworthy that when the incident angle θ varies from 0° to 70° for both TE and TM polarization, there are two significant plasmon resonance dips at 0.52 THz and 1.36 THz, which can coincide with the two vibration fingerprints of the lactose molecule in the spectrum. At the same time, it also shows that DAIGS is insensitive to incidence angles, which makes the structure have excellent molecular detection performance in a wide range of incidence angles.

**Figure 15:**
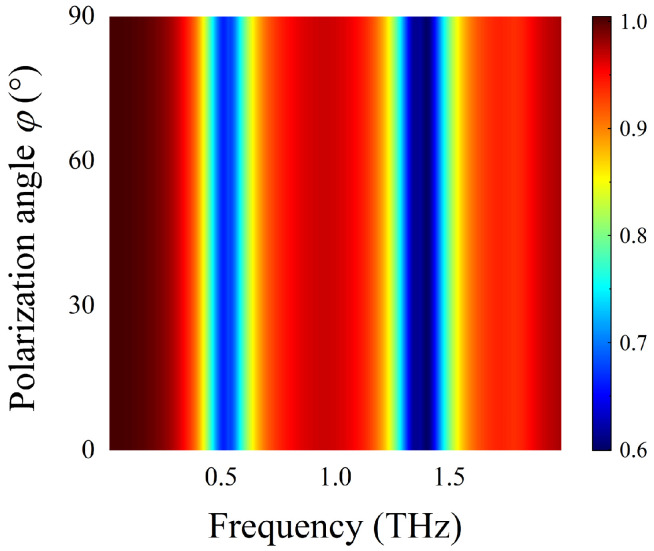
Transmission spectra of lactose-covered DAIGS at different polarization angle φ under the vertically incidence waves.

Finally, we investigate the dependence of detection performance of the DAIGS on the polarization angle φ of the incidence waves. Figure 15 shows the transmission spectra generated when an incidence wave with a polarization angle from 0° to 90° irradiated onto DAIGS under vertical incidence. It can be found that the transmission spectra hardly change with the polarization angles. When φ is 0°, the transmittance is consistent with the solid-line curve in Figure 13. When φ is 90°, the transmittance is consistent with the dashed curve in Figure 13. While as φ changes in the range of 0° to 90°, the transmission spectra of the structure are unchanged. The analysis shows that the vibrational fingerprint spectrum of lactose can be observed at any polarization angle.

The resonance frequency and amplitude of the DAIGS biosensor proposed in this paper are not affected by changes in polarization angle and incident angle, reflecting the characteristics of wide incidence angle and polarization insensitivity. In practical application, this feature can effectively eliminate the error of detection and make the measurement result more accurate.

## 5. Conclusions

The AIGS proposed in this paper has the advantages of fewer layers of dielectric materials, dynamically adjustable resonant frequency, insensitive to the incidence angle, and polarization direction. What is more, these two resonant frequencies can be tuned precisely to coincide with the two standard resonances of the lactose molecule. In addition, we studied the refractive index sensing characteristics of the proposed structure, and the sensitivity of the low-frequency and high-frequency transmission peaks can reach 0.035 THz/RIU and 0.10 THz/RIU, respectively, when the top layer is coated with different refractive index detections. Finally, we further design a quadruple rotationally symmetric DAIGS to solve the polarization sensitivity problem. We believe that the sensor structures designed in this paper will provide valuable guidance for graphene-based multi-resonance terahertz biosensors.

## Data Availability

The data that support the findings of this study are available from the corresponding author upon reasonable request.

## Abbreviations

The following abbreviations are used in this manuscript:

AIGS	Arc I-shaped graphene structure
DAIGS	Double arc I-graphene structure
SPPs	Surface plasmon polaritons
SPR	Surface plasmon resonance
LSPR	Local surface plasmon resonance
